# Development and Evaluation of a Component Level Implementation Fidelity Rating System for the GenerationPMTO Intervention

**DOI:** 10.1007/s11121-020-01177-5

**Published:** 2020-10-25

**Authors:** Kendal Holtrop, Debra L. Miller, Jared A. Durtschi, Marion S. Forgatch

**Affiliations:** 1 Department of Human Development and Family Studies, Michigan State University, 552 W. Circle Drive, East Lansing, MI 48824 USA; 2 School of Family Studies and Human Services, Kansas State University, Manhattan, KS USA; 3 Implementation Sciences International, Inc., Eugene, OR USA; 4 Oregon Social Learning Center, Eugene, OR USA

**Keywords:** Active ingredients, Component level fidelity, Component level implementation fidelity rating system, Component-specific fidelity, Fidelity, GenerationPMTO

## Abstract

Empirically determining the components of evidence-based interventions contributing to positive change is a crucial, yet understudied area of research. In support of this aim, we describe the development and evaluation of an observational rating system for measuring fidelity to specific components of the evidence-based GenerationPMTO parenting intervention. A five-step process was employed to systematically develop the rating system, which included consultation with the intervention developer and input from additional GenerationPMTO experts. The rating system was then tested using 247 h of video data from 184 parenting group intervention sessions. Study findings support the psychometric properties of the new measure with regard to item performance, reliability (i.e., inter-rater reliability of items, dimensionality of components, internal consistency of component scales), and validity (i.e., content validity, convergent validity, discriminant validity, and predictive validity of the component scales) for seven of the eight scales evaluated. The seven components include clear directions, skill encouragement, emotion regulation, limit setting, effective communication, problem solving, and monitoring. Data did not support the psychometric properties of the positive involvement scale. Overall, the ability to assess component-specific fidelity allows for a more nuanced examination of change processes, with meaningful implications for research and practice.

**Electronic supplementary material:**

The online version of this article (10.1007/s11121-020-01177-5) contains supplementary material, which is available to authorized users.

Empirically determining the components of evidence-based interventions responsible for achieving positive change is a crucial, yet understudied area of research (Abry et al. 2015; Blase and Fixsen 2013; Weisz and Kazdin 2017). The intervention testing process is too often limited to comparing average change between randomly assigned conditions; yet, the change process itself remains shrouded in a figurative “black box” (Abry et al. 2015; Nelson et al. 2012). This lack of understanding regarding how and why evidence-based interventions achieve positive change is a critical barrier impeding more effective translation of prevention programs into everyday practice (Spoth et al. 2013). To move science forward, we must evaluate intervention delivery at the component level and how each component is associated with key outcomes (Abry et al. 2015; Blase and Fixsen 2013).

To pursue this research agenda, it is necessary to develop fidelity measures capable of assessing distinct intervention components (Abry et al. 2015; Century and Cassata 2014; Nelson et al. 2012). Measuring fidelity, in general, is important for assessing the presence, dose, and/or quality of an intervention (Dusenbury et al. 2003; Mowbray et al. 2003). However, broad measures that only evaluate fidelity to an overall program fail to capture nuanced details of intervention delivery (Abry et al. 2015; Century and Cassata 2014). Despite prior calls to monitor implementation at the component level (Durlak and DuPre 2008), there remains a lack of empirical work developing and testing component-specific fidelity measures (Abry et al. 2015), and few such tools exist (Century and Cassata 2014). The goal of this study was to develop and evaluate an observational rating system for measuring fidelity to specific components of the evidence-based GenerationPMTO parenting intervention.

## Conceptual Framework for Implementation Fidelity

Implementation fidelity traditionally refers to the degree to which a program is delivered as intended by program developers (Dusenbury et al. 2003). It plays a central role in ensuring internal validity, allows for systematic assessment of intervention delivery to address important questions about why programs succeed or fail, and has been associated with better effects (Durlak and DuPre 2008; Dusenbury et al. 2003; Mowbray et al. 2003; Nelson et al. 2012). Implementation fidelity is a highly relevant lens for prevention research on dissemination and implementation (D&I) because it enables assessment of the degree to which evidence-based interventions are delivered as intended in real-world settings (Rabin and Brownson 2017; Spoth et al. 2013). This concept is encompassed within a number of D&I frameworks, including the highly influential conceptual framework for implementation fidelity (Carroll et al. 2007).

The conceptual framework for implementation fidelity provides a roadmap for measuring this important construct by organizing various fidelity domains into a coherent framework for empirical work (Carroll et al. 2007). According to the model, evaluating implementation fidelity is tantamount to measuring adherence—that is, how well the delivery of an intervention aligns with what was planned by its developers (Carroll et al. 2007). Adherence is operationalized as a multidimensional construct that takes into account intervention content, frequency, duration, and coverage. This framework also identifies several factors that can impact intervention delivery and therefore may serve as potential moderators of adherence, such as quality of delivery and participant responsiveness. We drew on the conceptual framework for implementation fidelity (Carroll et al. 2007) to inform our development of an implementation fidelity rating system.

## Component-Specific Fidelity Measurement

An issue at the forefront of fidelity scholarship is the importance of component-specific fidelity measurement. While not new to the literature (e.g., Durlak and DuPre 2008; Mowbray et al. 2003), this notion has become increasingly prominent. Many scholars now advocate for identifying and assessing core components as part of fidelity measurement, with more recent definitions of fidelity explicitly focusing on components (e.g., Century et al. 2010; Nelson et al. 2012). For instance, Nelson et al. (2012) define implementation fidelity as “the extent to which an intervention’s core components have been implemented…as planned” (p. 377).

Measuring implementation fidelity at the component level is an important step toward advancing knowledge of how evidence-based interventions operate, providing new opportunities to enhance prevention science. Currently, decision-makers must often rely on a theoretical understanding of core components when delivering interventions. Component level fidelity measurements allow for a data-driven examination of the extent to which each component relates to targeted outcomes, enabling critical intervention components to be determined with greater confidence (Abry et al. 2015; Century and Cassata 2014). This understanding can then fuel empirically informed decisions to optimize and adapt interventions. To optimize interventions, decision-makers could focus resources on the most potent components and consider trimming or revising non-essential parts (Abry et al. 2015; Blase and Fixsen 2013; Nelson et al. 2012). This could bring about briefer, more efficient, and cost-effective programs for delivery in real-world settings (Blase and Fixsen 2013; Michie et al. 2009). To guide intervention adaptation, decision-makers could use knowledge of critical components to identify areas of the program most suitable for adaptation to fit the local context (Carroll et al. 2007; Century et al. 2010; Durlak and DuPre 2008). A component-specific fidelity tool could also be useful for determining how much fidelity is sufficient for achieving intended outcomes (i.e., more is not always better), helping to identify components with more flexible levels of fidelity (Durlak and DuPre 2008; Nelson et al. 2012) as well as when lower fidelity due to contextually relevant adaptations may in fact improve outcomes (Century and Cassata 2014; Sanetti and Kratochwill 2009).

More broadly, this line of work may contribute to identifying critical components across different interventions and developing a more unified understanding of the change process (Abry et al. 2015; Century and Cassata 2014). For example, in the parenting intervention literature, advances are being made to understand the intervention elements associated with positive outcomes for children and families across various programs (e.g., Kaminski et al. 2008; Leijten et al. 2019; Melendez-Torres et al. 2019). However, this progress has been limited by a lack of information regarding component duration, dosage, intensity, and other treatment fidelity details (Kaminski et al. 2008; Melendez-Torres et al. 2019). The time is ripe for the development and evaluation of a fidelity rating tool to aid in component-specific fidelity measurement.

## GenerationPMTO

The focal parenting intervention for this rating system is GenerationPMTO (GenPMTO; Forgatch and Gewirtz 2017; Forgatch and Patterson 2010). GenPMTO, formerly known as Parent Management Training–the Oregon Model (PMTO®), is an evidence-based program delivered to caregivers to improve parenting practices and reduce coercive parent-child interactions. Based on more than 50 years of research, it is recognized as a well-established intervention for preventing and treating child behavior problems. GenPMTO has been successful in preventing delinquency and arrests for youth and improving maternal standard of living and arrest outcomes at 9-year follow-up (Forgatch et al. 2009; Patterson et al. 2010).

GenPMTO is characterized by five positive parenting practices that are considered its core components (Forgatch and Gewirtz 2017; Forgatch and Martinez 1999). *Skill encouragement* involves teaching children new skills through contingent positive reinforcement. *Limit setting* includes setting clear expectations and using mild, non-punitive discipline to reduce problem behaviors. *Problem solving* encourages structured and proactive family discussions for achieving family goals and addressing problems. *Monitoring* empowers caregivers to supervise children’s activities and interactions. Finally, *positive involvement* emphasizes the importance of showing children love and affection. GenPMTO also integrates additional support skills focused on clear directions, emotion regulation, and effective communication (Forgatch and Gewirtz 2017). Having such clearly articulated core components makes GenPMTO a strong candidate for development of a component level fidelity measure.

Furthermore, GenPMTO is an ideal target for fidelity research because of its established commitment to maintaining and investigating implementation fidelity. For example, after a temporary dip in fidelity between the first and second generations (Forgatch and DeGarmo 2011), high fidelity has been sustained for over 15 years during nationwide GenPMTO implementation in Norway (Askeland et al. 2019). Additionally, higher levels of fidelity to GenPMTO have been shown to predict greater improvements in parenting practices (Forgatch and DeGarmo 2011; Forgatch et al. 2005) and child problem behaviors (Hukkelberg and Ogden 2013).

These combined factors represent a valuable opportunity to advance measurement innovation in the area of component-specific fidelity and ultimately enhance understanding of intervention components contributing to positive change.

## The Current Study

The purpose of this study was to develop and evaluate an observational rating system for measuring component-specific fidelity of an evidence-based parenting intervention. Specifically, this rating system assesses adherence to individual components of GenPMTO. The current study contributes to the literature in a number of ways. First, it describes how a component level implementation fidelity rating system was conceptualized and developed. This helps fill the gap in the literature related to how component-specific fidelity measures can be created and utilized (Abry et al. 2015). Second, it introduces a component level implementation fidelity measure for assessing fidelity to distinct components of the GenPMTO intervention and describes its initial psychometric properties. To date, few tools exist for measuring fidelity with this degree of specificity (Century and Cassata 2014). Finally, this study can stimulate continued research to empirically identify active ingredients of evidence-based interventions, leading to a better understanding of how they achieve positive change. This is a critical step toward more effectively translating evidence-based interventions into everyday service settings to enhance their public health impact (Blase and Fixsen 2013; Gottfredson et al. 2015; Spoth et al. 2013).

## Method

### Data Source

This study utilized video data collected as part of a NIH-funded prevention study examining the effectiveness of the GenPMTO intervention over time (see Forgatch and DeGarmo 1999; Forgatch et al. 2009). In the original study, families were randomly assigned to either GenPMTO or a no-treatment control. Participation in the GenPMTO condition took place in a group format across 14–16 weekly sessions attended by the child’s caregiver. For the purposes of the current study, video data were available from 14 parenting group cohorts (i.e., 13 standard intervention groups plus one pilot testing group). The original video data were recorded on VHS tapes and then professionally digitized prior to use in this study.

### Participants

A total of 238 recently separated mothers and their school-aged sons (grades 1–3) participated in the original study. At baseline, mothers had been separated for an average of 9.2 months. Most mothers had some academic or vocational training beyond high school (76%), although only 17% had completed a 4-year college degree or higher. Mothers’ mean age was 34.8 years (SD = 5.4; range = 21.4 to 49.6). Boys’ mean age was 7.8 years (SD = .93; range = 6.1–10.4). The racial/ethnic composition of the boys in the sample was 86% White, 1% African American, 2% Latino, 2% Native American, and 9% other or multiracial. This reflected the racial/ethnic distribution of the community in which the study was conducted. The mean annual family income was $14,900, and 76% of families were receiving public assistance. The video data for the current study were recorded during the GenPMTO sessions attended by the intervention group. This included a subsample of 157 participants. Demographic data were missing from four families. The intervention and control groups did not differ on any baseline variables except months since separation and boys’ age. Intervention group mothers had been separated an average of 9.84 months; intervention group boys had an average age of 7.65 years.

### Interventionists

Group sessions were co-led by two interventionists, with eight in total delivering the intervention. They varied in educational training: three had a PhD, two had a master’s degree, one had some college education, and two had a high school diploma. Experience using the parent training model ranged from 0 to 20 years. All were female. Those without previous experience in the model participated in a 2 to 4-month structured training program.

### Intervention

Participants in the intervention condition were enrolled in Parenting through Change (PTC; Forgatch 1994). PTC is the group-based version of GenPMTO, which includes the five core components (i.e., skill encouragement, limit setting, problem solving, monitoring, positive involvement) along with auxiliary support skills. PTC is a preventive intervention with a manualized curriculum (Forgatch 1994); it was originally delivered in 16 sessions (groups 1–5) and was subsequently condensed into a 14-week format (groups 6–14). PTC sessions last approximately 90 min and include three parts: (a) review and troubleshoot prior material, (b) introduce and rehearse a new topic, and (c) assign a home practice assignment (Forgatch 1994).

### Development of the Rating System

A five-step process (see McLeod and Weisz 2005) was employed to systematically develop the observational rating system in this study.

Step 1. **Rating system focus**. The GenPMTO literature delineates specific components that characterize the model (e.g., Forgatch and Gewirtz 2017; Forgatch and Patterson 2010), allowing us to identify focal components for fidelity assessment (see Schoenwald et al. 2011). Specifically, this rating system was developed to measure eight GenPMTO components: (a) clear directions, (b) skill encouragement, (c) emotion regulation, (d) limit setting, (e) effective communication, (f) problem solving, (g) monitoring, and (h) positive involvement. Guided by the conceptual framework for implementation fidelity, which identifies adherence as the central construct in fidelity measurement (Carroll et al. 2007), we focused our measurement efforts on assessing adherence to each component. A critical feature of this rating system is that, in contrast to fidelity tools meant to evaluate adherence to prescribed session content, *the current measure is able to quantify the extent to which each intervention component was delivered with fidelity across the course of the intervention*. As a result, this rating system can be used to advance current research by determining how much exposure to each intervention component a participant received and how exposure to each component is linked to outcomes.
Step 2. **Scale and item development**. In accordance with standard psychometric procedures (see Lambert and Hill 1994) and following the work of McLeod and Weisz (2005), we began by reviewing the GenPMTO literature to establish a strong basis for validity. This included program manuals, the GenPMTO fidelity protocol, empirical studies, and other sources describing the intervention components (e.g., Dishion et al. 2016; Forgatch 1994; Knutson et al. 2019). From this review, we established a preliminary set of items describing the content of each intervention component. Each item was then operationalized via a set of indicators that described how the item could be observed during a session (e.g., see Nelson et al. 2012). Next, we distributed the items for review to the intervention developer and a group of GenPMTO experts to obtain systematic feedback. After subsequent refinements to the items and component scales, the resulting product was reviewed and confirmed by the intervention developer.
Step 3. **Scoring strategy**. We determined ratings would occur at the macroprocess level (McLeod et al. 2013) with the intervention session as the unit of measurement. In line with other fidelity scholars (e.g., Breitenstein et al. 2010), we believe rating entire sessions will allow for the most comprehensive measurement of intervention adherence. Therefore, each item is rated on a 7-point Likert scale (0 *= not at all* to 6 *= extensively*) according to the extent that behavior was present during session. This rating accounts for both thoroughness and frequency of delivery (Hogue et al. 1994, 1996). Thoroughness attends to the breadth and comprehensiveness of delivery, whereas frequency considers the duration and number of times an item was addressed. Together these complementary dimensions combine to form an extensiveness rating for each item (Hogue et al. 1994, 1996). Extensiveness ratings have a well-established history in treatment integrity research and confer advantages over rating systems that only assess for presence or absence of an item (Waltz et al. 1993). Extensiveness ratings have been used successfully in prior studies (e.g., McLeod and Weisz 2010; Southam-Gerow et al. 2016) and are a proven design feature for observational assessments of treatment (McLeod et al. 2009).
Step 4. **Pilot testing and refinement**. Next, the principal investigator and a graduate research assistant used the measure to independently rate approximately 10% (*n* = 18) of the sessions. During pilot testing, the researchers discussed the clarity and utility of the items and their indicators to inform further refinements to the rating system. Intraclass correlation coefficients (ICC) were calculated at both the component and item levels using IBM SPSS Statistics version 24 based on a single measures, absolute agreement, two-way random effects model. These ICC(2,1) values were moderate to excellent (*r* = .55 to .92; Koo and Li 2016) for each of the eight component scales, providing initial support for their reliability. At the item level, 15 items demonstrated poor reliability (ICC < .50; Koo and Li 2016) and were re-evaluated and revised when necessary. This process resulted in the initial version of the Component Level Implementation Fidelity Rating System (CLIFRS), a 76-item measure that assesses adherence to eight GenPMTO components. The CLIFRS is included as Supplemental File A. A rating manual was also completed to accompany the CLIFRS (Holtrop et al. 2019).
Step 5. **Research application**. We subsequently evaluated the initial psychometric properties of the CLIFRS using video data from the prior GenPMTO prevention trial. All study procedures were approved by the Michigan State University institutional review board.

#### Raters

The six-person rating team consisted of the principal investigator, two doctoral students, and three undergraduate students (one additional undergraduate student failed to achieve reliability and did not contribute rating data). The rating team ranged in age from 21 to 47 years (*M* = 27.7; SD = 9.3), identified as Caucasian (*n* = 5) or Latina (*n* = 1), and were all female. The principal investigator and one of the doctoral students, who served as the project manager, led the development and pilot testing of the rating system and were considered lead raters. The other raters were recruited and trained specifically for this project.

#### Rater Training

The first stage of training included experiential immersion in GenPMTO, assigned readings, reviewing the intervention manual, didactic instruction on the CLIFRS rating system, and practice rating videotaped sessions. These activities took place over 15 weeks and covered all eight intervention components. In the second stage of training, team members began rating videos. Each rater was assigned two videos each week, and ICCs were calculated to assess for inter-rater reliability with a lead rater. This training process continued until the rater had (a) rated a minimum of 6 sessions and (b) achieved an average ICC (1,1) > 0.70 with a lead rater. At this point, the rater was able to start rating independently (with regular inter-rater reliability checks). All training activities were led by the principal investigator or project manager.

#### Rating Assignment Plan

We sought to rate every weekly session of the PTC intervention groups (Forgatch and DeGarmo 1999). Prior parenting intervention research has emphasized the importance of rating all sessions, in their entirety, to ensure comprehensive assessment and capture variations in fidelity across time points and sessions (Breitenstein et al. 2010). Raters were assigned an average of two to four sessions each week, selected from different parenting groups to protect against spillover (“halo”) effects. All raters were naive to group-level outcomes. Reliability checks were performed regularly to monitor for rater drift, and bi-weekly meetings were held to view videos and provide continued training (see McLeod et al. 2013).

Out of the total 206 treatment sessions in the original effectiveness trial, we were able to rate 184 total sessions (89%). Data were missing from 18 sessions, damaged (i.e., no audio) from 3 sessions, and incomplete (< 45 min recorded) for one session. Overall, for this study, we rated approximately 247 h of video data. The total rating time, not including reliability training, was 380 h. On average, it took 105 min for a researcher to rate a video session.

### Additional Measures

#### Therapist Session Ratings Form

During the original GenPMTO trial, interventionists completed a therapist session ratings form for each group session to provide an evaluation of that session, report their affective states, track time spent on various process dimensions, and record how much of the intended curriculum was covered. For a subset of group sessions (*n* = 70), interventionists were asked to report the amount of the session spent on specific curriculum topics, with response options ranging from 0 (*none*) to 5 (*most of the session*). To arrive at session-level data, reports from both co-leaders were averaged prior to data analysis.

#### Parenting Practices

During the original study, multiple method assessments were conducted which included structured interviews and laboratory observations of 45-min parent-child interaction tasks. These assessments were scored using a well-established microsocial coding measure and global rating system to derive parenting practice measurements (see DeGarmo et al. 2004; Forgatch et al. 2009). For the current study, data from these structured interaction tasks were used to create composite indicators to measure skill encouragement (9 items; *α* = .79, .81, .66), emotion regulation (3 items; *α* = .84, .84, .75), inept discipline (13 items; *α* = .92, .92, .92), effective communication (3 items; *α* = .76, .74, .68), and problem solving (27 items; *α* = .93, .93, .95) at three time points. For monitoring, one observation-based item was combined with three interview items to derive a composite variable (4 items; *α* = .66, .68, .53). Negative reciprocity was based on behavior observed during the entire 45-min period as reported previously (Forgatch et al. 2009).

## Results

### Item Performance

We examine descriptive statistics to evaluate item performance (see Supplemental File B). This included checking the range of each item to ensure items were functioning as intended and had sufficient variation. The majority (88%) of items had a range of 4 or greater on the 0 to 6 scale, indicating adequate range. We also inspected item means and distributions. Item means ranged from 0.07 to 1.39 with overall *M* = 0.52 and SD = 0.27. In line with other established measures of treatment fidelity (e.g., Southam-Gerow et al. 2016), items were positively skewed.

### Reliability

#### Inter-Rater Reliability of the Items

Inter-rater reliability was calculated on a subsample of 20% (*n* = 36) of sessions. Two raters provided data for each reliability check, and rating pairs varied session-by-session. ICC estimates and their 95% confidence intervals were calculated using IBM SPSS Statistics version 24 based on a one-way random effects model, using a stringent standard of single-rating, absolute agreement (Koo and Li 2016; Shrout and Fleiss 1979). The ICC(1,1) data reported in Supplemental File B indicate moderate to good mean levels of inter-rater reliability (Koo and Li 2016) for the items comprising seven of the eight scales: clear directions (*M* = .89), skill encouragement (*M* = .81), emotion regulation (*M* = .75), limit setting (*M* = .78), effective communication (*M* = .71), problem solving (*M* = .89), and monitoring (*M* = .75). The reliability for one of the monitoring items could not be calculated due to zero variance. For the positive involvement scale, items demonstrated poor inter-rater reliability (*M* = .38). Overall, the average ICC across all 76 items was .73 (SD = 0.25). These data provide support for the inter-rater reliability of the items for each scale, with the exception of positive involvement.

#### Dimensionality of the Components

To identify if the items within each scale constituted a unidimensional construct, we first tested exploratory factor analyses (EFA) to evaluate how well the items fit together within each hypothesized component. We then tested confirmatory factor analyses (CFA) for each component as a latent variable, with the items that were identified as one factor in the EFA as indicators, to assess how well one underlying latent construct fit the data. Clear directions, skill encouragement, emotion regulation, limit setting, effective communication, problem solving, and monitoring all followed a similar pattern of results: The last item (i.e., reviewing home practice) was indicated as a second distinct factor in each of the EFA models. The EFA results for limit setting also showed that the seventh item did not fit as one factor with the others and it was dropped from subsequent analysis. All CFA models for these seven components had excellent fit to the data, with the exception of monitoring, which had only acceptable fit, in part due to the model being unable to converge with any additional error correlations: *χ*^2^ (16) = 38.76, *p* < .05, RMSEA = .09, CFI = .99, SRMR = .03. However, this was likely more a function of model complexity relative to sample size than a poor fitting model.

The remaining component, positive involvement, indicated multidimensional items that were not internally consistent and did not indicate acceptable model fit in the CFA analyses. Positive involvement is theorized as a distinct component of GenPMTO, but in practice, its content is interwoven throughout the other components. In further exploratory analyses, we decided to test the relevant positive involvement item within each other component scale. For instance, we tested if the positive involvement item related to the clear directions component would fit with the other clear directions items. This model demonstrated good fit: *χ*^2^ (14) = 26.97, *p* < .05, RMSEA = .07, CFI = .99, SRMR = .02. In fact, when testing EFA and CFA models for each of the other component scales, the embedded positive involvement item demonstrated very high standardized factor loadings above .70. This warrants further study in future research.

#### Internal Consistency

Informed by these factor analyses results, we next tested internal consistency at the component level using two indices of reliability. These results are included in Table 1. First, we calculated Cronbach’s alpha values. Higher alphas indicate greater internal consistency, with an alpha above .70 considered acceptable (Nunnally and Bernsetein 1994). The alphas for seven of these components were each excellent (*α*’s between .88 and .94). However, the alpha for positive involvement was only .44. Second, because alpha values are highly dependent on the number of scale items, we also calculated average inter-item correlations at the component level. Higher values indicate greater internal consistency, with an average inter-item correlation between .15 and .50 considered acceptable (Clark and Watson 1995). The average inter-item correlations for these components followed a similar pattern, where seven of the components had strong average inter-item correlations (i.e., .49 or greater). However, the average inter-item correlation for positive involvement was lower but still acceptable (*r* = .22).

**Table 1:** Component scale correlations and internal consistency statistics (*N* = 184 sessions)

Variables	1	2	3	4	5	6	7	8
1. Clear directions	-							
2. Skill encouragement	.11	-						
3. Emotion regulation	− .18*	− .04	-					
4. Limit setting	.32**	.12	− .12	-				
5. Effective communication	− .26**	− .21**	− .04	− .15*	-			
6. Problem solving	− .22**	− .16*	− .11	− .14	.04	-		
7. Monitoring	− .18*	− .13	− .21**	− .12	− .01	− .06	-	
8. Positive involvement	− .13	.03	− .12	− .09	.19*	− .06	.09	-
*α*	.94	.90	.88	.94	.90	.94	.92	.44
Average inter-item *r*	.71	.55	.49	.65	.59	.70	.62	.22

Component scale calculations were computed based on items indicated from the EFA results (clear directions items 1–7; skill encouragement items 1–8; emotion regulation items 1–9; limit setting items 1–6, 8, 9, 10; effective communication items 1–7; problem solving items 1–8; monitoring items 1–9, positive involvement items 8, 9, 10). Average inter-item *r* refers to the average inter-item correlation of the items in this component

*p < .05. **p < .01 (two-tailed)

### Validity

#### Content Validity

The systematic process used to develop the items in each rating scale provides strong support for the content validity of the CLIFRS. According to Lambert and Hill (1994), building on prior conceptualizations of the content of interest and obtaining expert feedback are two primary strategies for establishing content validity. As described previously, our item development process included a thorough review of the GenPMTO literature, obtaining feedback from GenPMTO experts, and collaborating with the intervention developer throughout this process. This methodical process provides strong support for the notion that the items comprising each scale adequately represent each parenting component.

#### Convergent Validity

We examined convergent validity of the observed component scores by assessing if they were related to interventionists’ self-reports of the content they delivered in session. We did this by evaluating correlations between the CLIFRS component scale scores and the data provided by interventionists on the therapist session ratings form regarding how much of the session was spent on different curriculum topics. Data were available for a subset of sessions (*n* = 70) that allowed for evaluation of validity for six of the component scales. Results support the convergent validity of the rating scale, with significant, strong, positive correlations between observed and self-reported content covered for each component examined: skill encouragement (*r* = .63, *p* < .01), emotion regulation (*r* = .82, *p* < .01), limit setting (*r* = .79, *p* < .01), problem solving (*r* = .67, *p* < .01), and monitoring (*r* = .78, *p* < .01).

#### Discriminant Validity

We examined discriminant validity to assess if the components were each statistically unique and distinct from one another. First, we calculated correlations among the component scale scores. A low correlation between two components would provide evidence to support discriminant validity. Examining the absolute values of these 28 correlations, only five are greater than .20 and none exceeded .32 (see Table 1). These low correlations provide initial support for discriminant validity of the rating scale components. We then went on to more precisely assess discriminant validity using chi-square difference tests applying a CFA approach. For example, the zero-order correlation of limit setting with clear directions was *r* = .32. We tested if the items within limit setting and clear directions fit significantly better as one total latent construct or as two distinct latent constructs. The two-factor model fits the data significantly better than a one-factor model, Δ*χ*^2^ (1) = 1112.11, *p* < .001, indicating the two components are distinct. Thus, even the two most highly correlated components fit significantly better as two distinct constructs. All of the 28 possible component pairings followed this same pattern of chi-square difference test results, indicating they were each significantly unique and distinct. This provides strong evidence for the discriminant validity of these eight components.

#### Predictive Validity

As a final test, we examined if the component scores measured from the intervention session video data predicted observed parenting behavior on future assessment occasions. To do this, we first aggregated the component scores at the parenting group level (calculating component scales as noted in Table 1), to quantify how much of each GenPMTO component was delivered with fidelity to each parent. We then compared those scores to independent laboratory observations of parenting practices at three time points: 6 months (Time 1), 12 months (Time 2), and 30 months (Time 3) post-baseline.

The results of the predictive validity analyses are depicted in Supplemental File C. A number of significant correlations were established. At the Time 1 assessment, which occurred shortly after intervention completion, higher CLIFRS ratings on the limit setting component were significantly associated with better observed limit setting by the caregiver (i.e., lower levels of “inept discipline”; *r* = − .19, *p* < .05). Higher ratings of effective communication were also significantly and positively associated with observed effective communication (*r* = .15, *p* < .05). At Time 2, higher CLIFRS ratings for skill encouragement, emotion regulation, and monitoring during the intervention sessions were significantly correlated with higher caregiver scores on skill encouragement (*r* = .16, *p* < .05), emotion regulation (*r* = .19, *p* < .05), and monitoring (*r* = .18, *p* < .05), respectively. In addition, greater exposure to clear directions content during the parenting intervention was significantly associated with less likelihood of negative reciprocity between the parent and child (*r* = − .21, *p* < .05). Furthermore, at Time 3, parents who received greater exposure to the problem solving component during the intervention demonstrated significantly higher levels of problem solving behavior with their child (*r* = .18, *p* < .05). Taken together, these findings support the predictive validity of this rating system.

## Discussion

Issues related to conceptualizing and developing fidelity measures deserve more attention in the literature (Mowbray et al. 2003; O’Donnell 2008). This paper describes development of the 76-item Component Level Implementation Fidelity Rating System (CLIFRS). The CLIFRS stands out for its ability to measure fidelity to specific intervention components—theory-based program elements at the core of GenPMTO. This work was informed by the conceptual model for implementation fidelity (Carroll et al. 2007), followed an established five-step procedure (McLeod and Weisz 2005), and included input from the intervention developer and other GenPMTO experts. The CLIFRS was then evaluated using data from a prior GenPMTO trial.

Study findings support the psychometric properties of the CLIFRS in assessing seven components: clear directions, skill encouragement, emotion regulation, limit setting, effective communication, problem solving, and monitoring. An eighth component scale, positive involvement, was not supported. Average ICC values across the seven confirmed scales ranged from .71 to .89, indicating appropriate inter-rater reliability. These scales were also internally consistent and unidimensional—further indicating a high degree of reliability. The one exception was the last item in each scale, which did not fit well within a single-factor structure. Although this last item, reviewing home practice, did not fit with the other items, we consider it a key element of the intervention. Thus, we do not recommend its removal but instead propose it be used as a distinct variable assessing a unique and valuable part of GenPMTO: reviewing the home practice. In addition, evidence of convergent validity was demonstrated via strong, positive correlations between interventionist reports of what was covered in session and the components observed by the rating team. Discriminant validity was evident in the weak correlations between components and through confirmatory factor analysis. This rating system also exhibited notable predictive validity, showing that CLIFRS ratings of components delivered during the intervention group were significantly correlated with corresponding parenting behaviors independently measured through laboratory observations at future assessment occasions.

### Application of the CLIFRS

Prevention scientists have emphasized that identifying, measuring, and monitoring fidelity to core components are critical aspects of intervention research (Gottfredson et al. 2015). To date, such efforts have been limited by the scarcity of assessment tools capable of measuring fidelity at the component level (Abry et al. 2015; Century and Cassata 2014). Fidelity assessment has typically focused on confirming that the independent variable was delivered as intended or ensuring facilitators are adhering to the model (e.g., Mowbray et al. 2003; Sigmarsdóttir and Guðmundsdóttir 2013)—approaches that aggregate fidelity across components. In contrast, the CLIFRS assesses the extent to which *each component* of the intervention was delivered with fidelity. This will enable researchers to examine associations between exposure to discrete intervention components and participant outcomes—a crucial advancement for enabling the identification of active ingredients of intervention programs (Abry et al. 2015).

#### Advancing Parenting Intervention Science

Fidelity to GenPMTO has traditionally been monitored by the well-established Fidelity of Implementation (FIMP) Rating System (Knutson et al. 2019). FIMP uses data from 10-min video segments, sampled from sessions focused on two intervention components (i.e., skill encouragement and limit setting), to assess practitioners’ competent adherence to GenPMTO (Knutson et al. 2019). Although it is a valuable tool for measuring fidelity to the overall model, FIMP is not equipped to evaluate fidelity specific to each GenPMTO component. In this way, the CLIFRS adds to, and uniquely complements, the fidelity assessment capacity of GenPMTO by providing a means by which to systematically rate adherence to each distinct component of GenPMTO during intervention delivery.

This has important implications for advancing parenting intervention science. GenPMTO leads the way in research on mechanisms of change following intervention exposure (Forehand et al. 2014). GenPMTO prevention studies have demonstrated a complex interplay of change processes where, for example, early improvements in effective parenting help to prevent youth delinquency and arrests 9 years later (Forgatch et al. 2009; Patterson et al. 2010). With these distal outcomes well-established, a critical next step is to examine more proximal change processes, such as what took place during intervention delivery to trigger these initial improvements in parenting. Using the CLIFRS, research can now investigate how fidelity to distinct GenPMTO components is associated with changes in parenting practices. This will extend existing research beyond aggregate measures of fidelity (e.g., Forgatch and DeGarmo 2011; Forgatch et al. 2005) to a more nuanced understanding of the role of distinct components. Using GenPMTO as the focal model, researchers can now better open the “black box” and advance understanding of the active ingredients operating in evidence-based parenting programs.

#### Advancing Systematic Adaptation

The ability to assess component-specific fidelity is a valuable means for providing empirical data to inform intervention adaptations targeted to different cultures and contexts. Information about component-specific fidelity can aid in the identification of essential program components that should be delivered with fidelity as well as supporting components that may be more suitable for adaptation to the local population (Carroll et al. 2007; Century et al. 2010; Durlak and DuPre 2008). Such data can also be used to determine the actual level of fidelity that is needed to foster positive outcomes and to identify situations where a more flexible degree of fidelity is suitable (Durlak and DuPre 2008; Nelson et al. 2012; Sanetti and Kratochwill 2009). Moreover, as efforts are made to continue identifying active program ingredients, component-specific fidelity measures such as the CLIFRS can be an important tool for supporting continued efforts to adapt interventions with both effectiveness and cultural relevance (Castro et al. 2004; Parra Cardona et al. 2012).

### Limitations and Future Directions for Research

The CLIFRS exhibited sound psychometric properties for assessing seven components of GenPMTO but did not show adequate reliability and validity for the positive involvement scale. We subsequently found that including a relevant positive involvement item within each other component scale resulted in a strong factor loading. This raises an interesting, albeit tentative, possibility that positive involvement content—things that promote warmth in the parent-child relationship and enhance child self-esteem—may be better conceptualized as an integral part of each other component, rather than a separate and distinct element. Said another way, perhaps teaching about how each GenPMTO component promotes connection and well-being is part of adhering to the model. This is an area for continued research and theory development. At present, the positive involvement scale should be revised prior to use in subsequent research.

We tested 21 specific theory-informed correlations to examine predictive validity and found 8 significant associations (*p* < .05), which is more than expected by chance. However, future studies may wish to specifically adjust for multiple correlations. In addition, the intervention data used in this study were collected in the 1990s. Data from this early GenPMTO trial were targeted specifically because intervention delivery took place before a formal fidelity manual was developed. This allowed us to observe variation in the delivery of intervention components, which is necessary for the research to take place but rare to find among more recent intervention studies (Blase and Fixsen 2013). At the same time, this presents a potential limitation as notions of fidelity have shifted over time. The focus of fidelity, in GenPMTO and the broader intervention literature, presently involves a multifaceted understanding of the construct that takes into account such factors as content adherence and quality of delivery (Knutson et al. 2019; Nelson et al. 2012). Future research should apply the CLIFRS with more recent GenPMTO data and alongside the existing FIMP measure (Knutson et al. 2019) to further evaluate the measure and continue to advance our understanding of fidelity. The CLIFRS is resource-intensive in terms of the training and time required to obtain fidelity data. It is, therefore, not responsive to calls to develop more effective and efficient fidelity rating systems (Schoenwald et al. 2011). Through continued application and testing, it may be possible to determine a sampling strategy that will allow for more efficient assessment.

## Conclusion

The CLIFRS is innovative in its ability to measure component-specific fidelity. The use of this new assessment tool can successfully allow for a more nuanced examination of the extent to which each intervention component was delivered with fidelity within a program session—and ultimately, throughout the course of the intervention. This has the exciting potential to open up the “black box” and advance the field of prevention science by helping to better discern the active ingredients operating in evidence-based parenting programs.

## Electronic supplementary material

ESM 1 (PDF 2.41 mb)

ESM 2 (PDF 156 kb)

ESM 3 (PDF 117 kb)
