# Supplementary material for: Development and Evaluation of a Component Level Implementation Fidelity Rating System for the GenerationPMTO Intervention
Source: Prev Sci. 2020 Oct 25;22(3):288–98. doi: 10.1007/s11121-020-01177-5 (PMC8032561; doi:10.1007/s11121-020-01177-5)
Supplement: Supplementary file 1 — (PDF 2.41 mb) [file 11121_2020_1177_MOESM1_ESM.pdf]

Component Level Implementation Fidelity Rating System (CLIFRS)

Rating Cover Sheet

Instructions: For each GenerationPMTO intervention session, rate the extensiveness with which each item was addressed. When determining the extensiveness rating, take into account both the frequency and thoroughness with which the item occurred.

Extensiveness ratings will occur on a **7-point Likert scale ranging from 0-6**, with the following anchors:

0 = Not at all
2 = Somewhat
4 = Quite a bit
6 = Extensively

1. ITEM NAME. Indicators describing characteristic ways this item may be delivered and observed during the GenerationPMTO intervention session.

0 1 2 3 4 5 6

Not at all Somewhat Quite a bit Extensively

Rater Name:

Date of Rating:

Video File Name:

Time Stamp (session start):

Time Stamp (session end):

Session Topic:

Are any videos shown in the session: ☐ Yes ☐ No

If yes, time stamp for video(s):

NOTES (anything unusual, such as prior missed session due to weather, participants missing, new member added, fill in facilitator, etc.):

Time to code video (circle to nearest 10-minute mark):

60 70 80 90 100 110 120 130 140 150 160 170 180 Other:________

Detailed guidelines for use of the CLIFRS are provided in the CLIFRS manual:

Holtrop, K., Miller, D. L., & Forgatch, M. S. (2019). *Rating manual for the Component Level Implementation Fidelity Rating System (CLIFRS)* [Unpublished manual]. Department of Human Development and Family Studies, Michigan State University.

Development of the CLIFRS was supported by the Eunice Kennedy Shriver National Institute of Child Health & Human Development of the National Institutes of Health under Award Number R03HD091640. The content is solely the responsibility of the authors and does not necessarily represent the official views of the National Institutes of Health.

Evidence-based Parenting Intervention Components (EPIC) Research Study

CLEAR DIRECTIONS

1. VERBAL TEACH: Clear Directions Are A Tool For Increasing Cooperation. Giving clear directions increases likelihood of child cooperation; how parents give a direction affects child's response; clear directions are a tool for teaching cooperation/compliance

0 1 2 3 4 5 6

2. VERBAL TEACH: Cooperation = 70% Of Time, Within 10 Seconds. 100% compliance is not realistic, do not expect perfection; children who do well cooperate about 70% of time; give child time to cooperate = 10 seconds to get started; children may struggle to cooperate during stressful times

0 1 2 3 4 5 6

{PI1. VERBAL TEACH: Clear Directions Support The Parent-Child Relationship.} Using clear directions increases opportunities for positive child, parent responses and parent-child interactions; reduces negative exchanges and limit setting encounters; use positive tone; stay calm; be respectful; anticipate compliance

0 1 2 3 4 5 6

3. VERBAL TEACH: Characteristics Of Unclear Directions. These include: "drive by" directions, using too many words, nagging, being vague, showing hostility, being sarcastic, asking a question (Will you..?), threatening, yelling, and/or using "stop" commands

0 1 2 3 4 5 6

4. VERBAL TEACH: Characteristics Of Clear Directions. These include: get child's attention, say child's name, tell what TO do, make it short and simple, make a statement, be calm, be firm yet polite, give one direction at a time, pay attention to timing; stand and hold, follow through with praise

0 1 2 3 4 5 6

5. DEMONSTRATE: Clear Directions. Leaders provide verbal demonstrations of clear/unclear directions; act out role plays of "wrong way" and "right way" directions; clear directions typically include use of the phrase: "[child] do ------- now, please."

0 1 2 3 4 5 6

6. FACILITATE IN-SESSION PRACTICE: Clear Directions. Parents guided to practice clear directions in session, such as: having them shape a negative example into a clear direction during group discussion, through playing a game or an activity, using flashcards, or participating in a role play

0 1 2 3 4 5 6

7. ASSIGN HOME PRACTICE: Clear Directions. Specific home practice assignment (HPA) given to parents focused on clear directions; typically includes practice applying skills at home with their children; Ex: track use of clear directions and child responses, practice using clear directions

0 1 2 3 4 5 6

8. REVIEW HOME PRACTICE: Clear Directions. Checking in about trying out clear directions HPA; troubleshooting clear directions (even in cases when parents did not complete HPA); helping parents personalize tools for their use; Ex: reviewing tracking of clear directions and child responses, checking in about use of clear directions

0 1 2 3 4 5 6

Note: "Directions" were referred to in prior PMTO intervention work as "requests." In contemporary applications of GenerationPMTO, the term "clear directions" may be used synonymously with "good directions" or "effective directions." Raters should, therefore, allow for flexible terminology when scoring these items.

SKILL ENCOURAGEMENT

1. VERBAL TEACH: Skill Encouragement Is A Tool For Teaching Desired Behaviors. Paying attention to a behavior increases its frequency; encouragement teaches positive behaviors, cooperation, important life skills, routines (vs. punishment discouraging problem behavior)

0 1 2 3 4 5 6

2. VERBAL TEACH: Encourage Behaviors With Contingent Positive Reinforcement. How skill encouragement works=use contingent positive reinforcement to reward desired behaviors (“shine a light on what you want to grow”); focus on positives; when children cooperate provide praise/encouragement; **be consistent, follow through**; don’t take away earned incentives, no backhanded compliments; range from social reinforcers to tangible rewards

0 1 2 3 4 5 6

{PI2. VERBAL TEACH: Skill Encouragement Is A Positive Experience For Child/Strengthens Parent-Child Relationship.} Encouraging children develops confidence and self-esteem of child, strengthens the parent-child relationship; promotes a strengths-based atmosphere (vs. punishment); set children up to succeed; make encouragement fun; trust is built by providing earned rewards; be consistent; do not require perfection

0 1 2 3 4 5 6

3. VERBAL TEACH: Break Complex Behaviors Into Smaller Steps. Importance of breaking complex behaviors into manageable steps child can accomplish; help parents realize the complexity of everyday tasks; emphasize importance of praising small accomplishments along the way; encourage progress toward goal

0 1 2 3 4 5 6

4. VERBAL TEACH: Rationale For Incentive Charts. ***Reasons for*** incentive chart, such as: teach routines and complex skills, helps child succeed, provide consistency and clear expectations to child, focus parent attention on prosocial behavior; incentives reward positive behavior once it has occurred and are not bribes (charts aka “contracts”)

0 1 2 3 4 5 6

5. VERBAL TEACH: Instructions For Incentive Charts. ***How to*** set up/use incentive chart: identify goal behavior, break behavior into steps (5 steps), identify realistic incentives, assign points to steps (perfection not required; 70% compliance as goal), identify how to help child succeed, explain chart to child; use and review daily, troubleshoot; change goal behavior once learned (charts aka “contracts”)

0 1 2 3 4 5 6

6. DEMONSTRATE: Skill Encouragement. Leaders act out examples or role plays of skill encouragement strategies, such as: making an incentive chart, teaching and practicing goal behaviors with children, good way/ bad way to review incentive chart and provide rewards; leaders show parents how to make incentive chart

0 1 2 3 4 5 6

7. FACILITATE IN-SESSION PRACTICE: Skill Encouragement. Parents guided to practice skill encouragement in session, such as identify a goal behavior, break a complex behavior into smaller steps, identify relevant incentives, design an incentive chart; engage in role play explaining incentive chart to child

0 1 2 3 4 5 6

8. ASSIGN HOME PRACTICE: Skill Encouragement. Specific home practice assignment (HPA) given to parents focused on skill encouragement; typically includes practice applying skills at home with children; Ex: set up/use an incentive chart with child

0 1 2 3 4 5 6

9. REVIEW HOME PRACTICE: Skill Encouragement. Checking in about trying out skill encouragement HPA; troubleshooting skill encouragement (even in cases when parents did not complete HPA); helping parents personalize tools for their use; Ex: review and troubleshoot use of incentive charts

0 1 2 3 4 5 6

EMOTION REGULATION

1. VERBAL TEACH: Emotion Regulation Is A Tool For Enhancing Effective Parenting. Ability to identify and regulate emotions is prerequisite to all PMTO parenting skills; need to use emotion regulation to stay calm, avoid escalation, keep conflicts from getting out of control, prevent “rubber band conflicts” and coercion

0 1 2 3 4 5 6

2. VERBAL TEACH: Emotions Can Have A Powerful Effect On Others. Parent emotions can impact child emotions and behaviors; negative emotions can disrupt parenting; emotions can impact others around us; influence of emotions on relationship with other family members/parent [general impacts, not specific to p-c relationship]

0 1 2 3 4 5 6

{PI3. VERBAL TEACH: Emotions Can Interfere With Parent-Child Relationship.} Specific focus on emotions helping or harming parent-child relationship; staying calm, showing positive/neutral emotions (ex: interest, empathy) good for parent-child relationship; Lack of ER (ex: chronic anger, depression) can damage parent-child relationship

0 1 2 3 4 5 6

3. VERBAL TEACH: It Is Important To Be Able To Identify Emotions. Must be able to recognize/ identify/observe emotions; parents and children may experience different emotions (ex: parent is angry, child is afraid; parent is depressed, child is worried); need to differentiate own emotions from children

0 1 2 3 4 5 6

4. VERBAL TEACH: Characteristics Of Different Emotions. Explaining characteristics of different emotions, ways to identify different emotions, such as facial expression, tone of voice, body posture (ex: depression = sad face, slumped body; gloomy outlook); how to identify emotions in self and children

0 1 2 3 4 5 6

5. VERBAL TEACH: It Is Possible To Regulate Emotions. It is possible to manage/regulate your emotional expression, change how you feel; many people are not taught about emotion regulation; parents can control their emotions; avoid too much/too little emotional control; possible to “turn up” or “turn down” emotions

0 1 2 3 4 5 6

6. VERBAL TEACH: Techniques For Regulating Emotions. There are many strategies for managing emotions, such as: disengage (walk away), listen to music, think of a happy place, focus on goal, take deep breaths, use humor, etc.; may include looking at handout for ideas

0 1 2 3 4 5 6

7. DEMONSTRATE: Emotion Regulation. Leaders act out examples or role plays about identifying or regulating emotions; demonstrate examples of emotional expression (ex: depressed, angry, interested) or emotion regulation strategies; use visual aids to illustrate different emotions

0 1 2 3 4 5 6

8. FACILITATE IN-SESSION PRACTICE: Emotion Regulation. Parents guided to practice identifying or regulating emotions through role plays, discussion of visual aids, brainstorming, or activities; engage in emotion regulation exercises (ex: guided breathing); practice differentiating parent vs. child emotions

0 1 2 3 4 5 6

9. ASSIGN HOME PRACTICE: Emotion Regulation. Specific home practice assignment (HPA) given to parents focused on identifying and/or regulating emotions; typically includes practice applying skills at home with their children; Ex: observe, identify, manage, and/or track emotions; track emotions while creating a collage

0 1 2 3 4 5 6

10. REVIEW HOME PRACTICE: Emotion Regulation. Checking in about trying out identifying and/or regulating emotions HPA; troubleshooting identifying and regulating emotions (even in cases when parents did not complete HPA); helping parents personalize tools for use with their families; Ex: review emotions that were tracked during the week; review emotions that were tracked while creating a collage

0 1 2 3 4 5 6

Note: In many versions of PMTO, emotion regulation is taught as two consecutive sessions: observing emotions and managing emotions. Both of these topics are covered within the Emotion Regulation component.

In prior applications of PMTO, “regulating emotions” is often referred to as “managing emotions” or “controlling emotions.” Raters should allow for flexible terminology when scoring past iterations of PMTO.

LIMIT SETTING

1. VERBAL TEACH: Limit Setting Is A Tool For Decreasing Problem Behaviors. Discipline is used to discourage or decrease frequency of problem behaviors (vs. encouragement increasing positive behavior), set limits, keep children safe, provide structure; puts parent (not child) in charge; discipline is a teaching tool

0 1 2 3 4 5 6

2. VERBAL TEACH: Apply Mild, Negative Consequences. How limit setting works=give mild, negative consequences for misbehavior; **be consistent and follow through**; avoid "shining light" on misbehavior (negative attention can increase misbehavior); avoid too strong/too weak parental reactions (need right amount for child to learn); best to use consistent, small, immediate consequences; sandwich metaphor; avoid threats; consequences ≠ threats

0 1 2 3 4 5 6

{PI4. VERBAL TEACH: Limit Setting Can Be Accomplished Without Harming The Parent-Child Relationship.} Essential to balance encouragement with discipline = 5:1 ratio; need limit setting + warmth; make clear to child you discipline behavior but still love them; keep discipline mild, short, safe for child; clear rules and predictable consequences; don't hold grudges (when it's over, it's over); discipline ≠ revenge; stay calm, use emotion regulation

0 1 2 3 4 5 6

3. VERBAL TEACH: Rationale For Time Out. ***Reasons for*** TO: brief, mild consequence; child sent to neutral setting to disengage, stop misbehavior, calm down; TO avoids giving attention to misbehavior; prevents escalation, facilitates emotion regulation; helps child learn to make good decisions; helps parents feel prepared, calm, confident

0 1 2 3 4 5 6

4. VERBAL TEACH: Instructions For Time Out. ***How to*** use TO sequence: give clear direction (CD); stand and hold; if noncomply, repeat CD or go to TO; if refusal, label misbehavior, send to TO (5 min); if refusal, increase TO by 1 min up to 10 min; if refusal, go to TO or privilege removal (PR); if refusal, PR; disengage to avoid escalation (when it's over, it's over); stay calm, use slow pace; how to ID appropriate TO locations, prepare TO, and explain to child

0 1 2 3 4 5 6

5. VERBAL TEACH: Rationale For Privilege Removal. ***Reasons for*** privilege removal: mild consequence that "costs" slightly more than TO where a privilege or possession is briefly removed; does not require child compliance; typically used as backup for TO refusal, but can also be used alone; helps children learn choices have consequences

0 1 2 3 4 5 6

6. VERBAL TEACH: Instructions For Privilege Removal. ***How to*** do PR: should be short (15-30min), take place soon after encounter, something parent is willing and able to remove (but not earned incentives or comfort toy/blanket); for older children/more serious offenses PR can be longer, used as backup to work chore; how to prepare for PR

0 1 2 3 4 5 6

7. VERBAL TEACH: Other Limit Setting Tools. Explain work chore (WC) and/or fines; **WC**: 5-15 minute chore as consequence for misbehavior (for older children/more severe behavior WC may be longer and split up into several daily tasks); **Fines**: portable consequence used in the moment for mild misbehavior (ex: $0.25 fee)

0 1 2 3 4 5 6

8. DEMONSTRATE: Limit Setting. Leaders act out examples or role plays showing TO sequence for a number of different end points, use of privilege removal, work chores and/or fines; role plays demonstrating how to introduce children to new limit setting practices

0 1 2 3 4 5 6

9. FACILITATE IN-SESSION PRACTICE: Limit Setting. Parents guided to practice limit setting skills in session, such as engaging in role plays, games, or activities about TO or PR; parents prepare for limit setting, such as identify TO locations, PR items, work chores; parents role play explaining new limit setting practices to children

0 1 2 3 4 5 6

10. ASSIGN HOME PRACTICE: Limit Setting. Specific home practice assignment (HPA) given to parents focused on limit setting; typically involves practice applying skills at home with children; Ex: ID time out location, plan privileges to remove, introduce time out to child, use time out if/when needed

0 1 2 3 4 5 6

11. REVIEW HOME PRACTICE: Limit Setting. Checking in about limit setting HPA; troubleshooting limit setting skills (even in cases when parents did not complete HPA); helping parents personalize tools for their use; Ex: review selected time out locations, privileges to remove; troubleshoot use of TO and PR with child

0 1 2 3 4 5 6

EFFECTIVE COMMUNICATION

1. VERBAL TEACH: Effective Communication Is A Tool For Exchanging Information. Being able to listen and speak effectively useful for exchanging information with others (ex: child, co-parent, other adults); need give and take of information; effective communication can strengthen relationships [but do NOT code this item for p-c relationship]

0 1 2 3 4 5 6

{PI5. VERBAL TEACH: Effective Communication Nourishes The Parent-Child Relationship.} The parent-child relationship grows with regular, friendly conversations; use active listening, show interest, be non-critical, show child their opinion is valued, regulate emotions, don't lash out; enjoy laughing together

0 1 2 3 4 5 6

2. VERBAL TEACH: Characteristics Of Effective Communication. Involves characteristics of effective communication **in general**: take turns, change roles (speaker and listener); respond, don't react; use emotion regulation skills; try to understand other's perspective; DON'T: be defensive, take over with talking, attack

0 1 2 3 4 5 6

3. VERBAL TEACH: Characteristics Of Active Listening. Involves characteristics **specific to** active listening: hear message accurately and paraphrase; set aside own feelings (emotion regulation); pay attention, make eye contact, show empathy, use good nonverbals; DON'T: interrupt, assume, interrogate, tune out, fix problem (unless asked)

0 1 2 3 4 5 6

4. VERBAL TEACH: Active Listening Helps Parents Monitor Children. Listening to children talk about activities and interests helps parents know what is going on in their lives; importance of daily communication/debriefing; includes active listening and contact with adults at children's school (ex: teachers) to **monitor school progress**

0 1 2 3 4 5 6

5. DEMONSTRATE: Effective Communication. Leaders act out examples of ineffective/effective communication, such as wrong way/right way role plays of when someone is not listening vs. listening; role playing common communication scenarios (ex: parent-child situation, communication with teacher)

0 1 2 3 4 5 6

6. FACILITATE IN-SESSION PRACTICE: Effective Communication. Parents guided to practice effective communication skills in session, such as distinguishing effective listening vs. ineffective listening using situation cards, practice good speaking skills, role playing different communication scenarios

0 1 2 3 4 5 6

7. ASSIGN HOME PRACTICE: Effective Communication. Specific home practice assignment (HPA) given to parents focused on effective communication; typically involves practice applying skills at home with children; Ex: engage in daily debrief with child, make collage with child using active listening skills

0 1 2 3 4 5 6

8. REVIEW HOME PRACTICE: Effective Communication. Checking in about effective communication HPA; troubleshooting effective communication skills (even in cases when parents did not complete HPA); helping parents personalize tools for their use; Ex: review daily debrief with child or active listening skills used while making collage

0 1 2 3 4 5 6

PROBLEM SOLVING

1. VERBAL TEACH: Problem Solving Is A Tool For Addressing Problems And/Or Achieving Goals As A Family. Useful when outcome is negotiable; all families encounter problems, need to set goals; problem solving can prevent future problems, reduce stress; ignoring/fighting about problems can intensify them; value of including all family members

0 1 2 3 4 5 6

{PI6. VERBAL TEACH: Problem Solving Prevents Conflict In The Parent-Child Relationship.} Avoid power struggles by involving child; allows child to participate in setting rules for their behavior; fosters willingness to cooperate with rules; provides process for rules to be revised, privileges updated; promotes atmosphere of mutual respect; method for problems to be addressed without yelling/fights

0 1 2 3 4 5 6

2. VERBAL TEACH: Rationale For Specific Problem Solving Procedure. ***Reasons for*** problem solving procedure: using these steps helps guide problem solving process, serves as strategy for making problems easier to solve, provides structure/safety; helps families work together, gives children voice in family matters, teaches responsibility and problem solving skills; non-blaming, focus on solutions; INCLUDES rationale for specific PS steps

0 1 2 3 4 5 6

3. VERBAL TEACH: Instructions For Specific Problem Solving Procedure. ***How to*** do the problem solving procedure: start with something positive, make a goal statement, brainstorm solutions, consider all solutions, combine/choose/make plan, write out agreement, try it out, revise as necessary

0 1 2 3 4 5 6

4. VERBAL TEACH: Rationale For Family Meetings. ***Reasons for*** holding family meetings: give structure for applying problem solving procedure as family; regularly scheduled opportunity to address problems, plan activities, make agreements, celebrate achievements as family; everyone can participate; INCLUDES rationale for specific FM steps

0 1 2 3 4 5 6

5. VERBAL TEACH: Instructions For Family Meetings. ***How to*** do family meetings: hold at regular time, limit duration (15-30 min), focus on one problem at a time, allow each person to talk, be polite, regulate emotions (calm, engaged), no phone calls or visitors, small consequence for not following rules

0 1 2 3 4 5 6

6. DEMONSTRATE: Problem Solving. Leaders act out examples or role plays of problem solving procedure and/or family meetings, such as actively modeling problem solving steps or using role play to demonstrate how to introduce family problem solving or family meetings to family

0 1 2 3 4 5 6

7. FACILITATE IN-SESSION PRACTICE: Problem Solving. Parents guided to practice problem solving procedure and/or elements of family meeting in session, such as engaging in specific steps to problem solving in group, using scenarios, or identifying family meeting rules for their use at home

0 1 2 3 4 5 6

8. ASSIGN HOME PRACTICE ASSIGNMENT: Problem Solving. Specific home practice assignment (HPA) given to parents focused on problem solving; typically includes practice applying skills at home with their children; Ex: hold a family meeting, use problem solving steps to plan a fun family event

0 1 2 3 4 5 6

9. REVIEW HOME PRACTICE: Problem Solving. Checking in about trying out problem solving HPA; troubleshooting problem solving activities (even in cases when parents did not complete HPA); helping parents personalize tools for their use; ex: troubleshoot use of family meeting, review problem solving steps used to plan fun family event

0 1 2 3 4 5 6

MONITORING

**1. VERBAL TEACH: Monitoring Is A Tool For Keeping Children Safe And/Or Out Of Trouble.** Keeping track of activities/behaviors helps keep children safe, out of trouble, engaged in appropriate activities; allows for early detection/intervention of problems; need consistent supervision, routines (inside and outside home)<br>0 1 2 3 4 5 6
**2. VERBAL TEACH: Children Need Different Levels Of Monitoring.** Different children/situations require different levels of monitoring, based on: child age (toddler vs. teenager), safety of location (in home, outside, community), responsibility level (child earns reduced supervision with demonstrated responsibility)<br>0 1 2 3 4 5 6
**{PI7. VERBAL TEACH: Monitoring Can Benefit The Parent-Child Relationship.}** Monitoring can encourage parents to spend time with children, can help build trust (ex: parent looks out for child safety, child demonstrates responsibility); parents can show involvement through support of child activities<br>0 1 2 3 4 5 6
**3. VERBAL TEACH: Aspects Of Monitoring Inside The Home.** Goal=*keep children safe, out of trouble*; Importance of monitoring what children are doing at home while parents are home or away (ex: at work); technology/media use and screen time; getting along with others at home (ex: siblings); what children are doing when they are alone<br>0 1 2 3 4 5 6
**4. VERBAL TEACH: Aspects Of Monitoring Outside The Home.** Goal=*keep children safe, out of trouble*; Importance of monitoring children away from home (ex: school, neighborhood); includes checking in routine, 5 key questions: where, who, what, when, how?; should know: friends, parents, contact info, transportation, safety concerns<br>0 1 2 3 4 5 6
**5. VERBAL TEACH: Involving Children In Skill-Building Activities.** Involving children in appropriate, structured activities can decrease unsupervised time (which means less chance to get into trouble) and promote skill development = worth the time and effort; includes athletics, music lessons, school activities, organized groups, etc.<br>0 1 2 3 4 5 6
**6. VERBAL TEACH: Protecting Children While In Care Of Others.** Importance of checking up on caregivers to ensure they are safe and reliable; includes childcare providers, babysitters, non-resident parents, coaches, etc.; can drop in, check references/reputation, note child behavior<br>0 1 2 3 4 5 6
**7. DEMONSTRATE: Monitoring.** Leaders act out examples or role plays of monitoring strategies or behaviors, such as doing check in routine with child or questioning a prospective childcare provider<br>0 1 2 3 4 5 6
**8. FACILITATE IN-SESSION PRACTICE: Monitoring.** Parents guided to engage in discussion and/or activity relevant to applying monitoring practices, such as: brainstorm check-in strategies, ideas for monitoring, extracurricular activities, neighborhood safety factors; make contact information list<br>0 1 2 3 4 5 6
**9. ASSIGN HOME PRACTICE: Monitoring.** Specific home practice assignment (HPA) given to parents focused on monitoring tools; typically includes practice applying skills at home with their children; Ex: make emergency contact list, use Parenting Resources Directory, internet, etc. to gather information; do check-in routine with child<br>0 1 2 3 4 5 6
**10. REVIEW HOME PRACTICE: Monitoring.** Checking in about trying out monitoring HPA; troubleshooting monitoring tools and strategies (even in cases when parents did not complete HPA); helping parents personalize tools for their use; ex: review phone lists/resources, troubleshoot check-in questioning process with child<br>0 1 2 3 4 5 6

POSITIVE INVOLVEMENT

PI8. VERBAL TEACH: Showing Children Affection. Importance of showing non-contingent love and affection to children; includes physical affection (hugs, high fives), verbal affection (telling child you love them), and other culturally appropriate forms; loving relationships promote healthy child development

0 1 2 3 4 5 6

PI9. VERBAL TEACH: Spending Time With Child. Importance of spending time with child; give child positive attention; ideas for fun family activities; play games or do other fun things with child; attend activities child is involved in (sports, clubs, recitals); find common interests or hobbies

0 1 2 3 4 5 6

PI10. VERBAL TEACH: Being Involved in Child's School Life. Showing child that you want them to experience school success and to enjoy school/educational activities; stay aware of child's school life; attend school functions and academic meetings; help child with homework; provide school-related encouragement; advocate for child at school

0 1 2 3 4 5 6

PI11. POSITIVE INVOLVEMENT NOT OTHERWISE SPECIFIED (NOS). Any instances where a leader teaches about positive involvement and ways parents can promote and/or show love in the parent-child relationship not covered within another PI item (ex: apologize to child when you do something wrong, take care of child when they are sick)

0 1 2 3 4 5 6
