# Supplementary material for: Development and Evaluation of a Component Level Implementation Fidelity Rating System for the GenerationPMTO Intervention
Source: Prev Sci. 2020 Oct 25;22(3):288–98. doi: 10.1007/s11121-020-01177-5 (PMC8032561; doi:10.1007/s11121-020-01177-5)
Supplement: Supplementary file 2 — (PDF 156 kb) [file 11121_2020_1177_MOESM2_ESM.pdf]

Supplemental File B

Item Performance Statistics

Item	Item Type	Brief Description	Range	*M*	*SD*	ICC	Lower CI	Upper CI
Clear Directions Scale								
CD1	Verbal Teach	Clear directions are a tool for increasing cooperation	6	0.66	1.13	0.83	0.70	0.91
CD2	Verbal Teach	Cooperation target is 70% of the time, within 10 seconds	5.5	0.47	1.09	0.92	0.85	0.96
CD3	Verbal Teach	Characteristics of unclear directions	6	0.47	1.12	0.78	0.61	0.88
CD4	Verbal Teach	Characteristics of clear directions	6	0.80	1.49	0.89	0.79	0.94
CD5	Demonstrate	Leader demonstrates clear directions material	6	0.61	1.23	0.86	0.74	0.93
CD6	Facilitate	Leader facilitates in-session application of clear directions material	6	0.87	1.80	0.89	0.80	0.94
CD7	Assign HP	Leader assigns home practice of clear directions material	6	0.45	1.12	0.94	0.88	0.97
CD8	Review HP	Leader reviews home practice of clear directions material	6	0.50	1.45	0.96	0.93	0.98
Skill Encouragement Scale								
SE1	Verbal Teach	Skill encouragement is a tool for teaching desired behaviors	3	0.55	0.81	0.67	0.45	0.82
SE2	Verbal Teach	Encourage behaviors with contingent positive reinforcement	6	1.30	1.37	0.66	0.43	0.81
SE3	Verbal Teach	Break complex behaviors into smaller steps	6	0.58	1.07	0.81	0.65	0.90
SE4	Verbal Teach	Rationale for incentive charts	6	0.89	1.19	0.68	0.46	0.82
SE5	Verbal Teach	Instructions for incentive charts	6	0.93	1.47	0.87	0.76	0.93
SE6	Demonstrate	Leader demonstrates skill encouragement material	6	0.52	1.14	0.89	0.80	0.94
SE7	Facilitate	Leader facilitates in-session application of skill encouragement material	6	0.49	1.20	0.87	0.76	0.93
SE8	Assign HP	Leader assigns home practice of skill encouragement material	4	0.60	0.89	0.85	0.73	0.92
SE9	Review HP	Leader reviews home practice of skill encouragement material	6	1.39	1.92	0.97	0.95	0.99
Emotion Regulation Scale								
ER1	Verbal Teach	Emotion regulation is a tool for enhancing effective parenting	6	0.57	0.94	0.42	0.03	0.70
ER2	Verbal Teach	Emotions can have a powerful effect on others	6	0.60	0.94	0.53	0.25	0.73
ER3	Verbal Teach	It is important to be able to identify emotions	4.5	0.61	1.10	0.90	0.80	0.95
ER4	Verbal Teach	Characteristics of different emotions	5	0.30	0.79	0.56	0.29	0.75
ER5	Verbal Teach	It is possible to regulate emotions	6	0.50	0.92	0.74	0.55	0.86
ER6	Verbal Teach	Techniques for regulating emotions	6	0.75	1.41	0.87	0.76	0.93
ER7	Demonstrate	Leader demonstrates emotion regulation material	6	0.40	0.93	0.71	0.50	0.84

ER8	Facilitate	Leader facilitates in-session application of emotion regulation material	6	1.31	2.24	0.97	0.95	0.99
ER9	Assign HP	Leader assigns home practice of emotion regulation material	4.5	0.56	1.08	0.86	0.74	0.93
ER10	Review HP	Leader reviews home practice of emotion regulation material	6	0.90	1.90	0.95	0.90	0.97
Limit Setting Scale								
LS1	Verbal Teach	Limit setting is a tool for decreasing problem behaviors	6	0.76	1.18	0.79	0.63	0.89
LS2	Verbal Teach	Apply mild, negative consequences	6	1.05	1.63	0.75	0.51	0.88
LS3	Verbal Teach	Rationale for time out	6	0.58	1.22	0.89	0.79	0.94
LS4	Verbal Teach	Instructions for time out	6	0.62	1.45	0.91	0.83	0.95
LS5	Verbal Teach	Rationale for privilege removal	5	0.33	.81	0.53	0.25	0.73
LS6	Verbal Teach	Instructions for privilege removal	5	0.46	1.10	0.67	0.44	0.82
LS7	Verbal Teach	Other limit setting tools	4	0.22	.67	0.79	0.63	0.89
LS8	Demonstrate	Leader demonstrates limit setting material	6	0.47	1.17	0.47	0.17	0.69
LS9	Facilitate	Leader facilitates in-session application of limit setting material	6	0.49	1.32	0.92	0.84	0.96
LS10	Assign HP	Leader assigns home practice of limit setting material	5	0.40	.96	0.88	0.78	0.94
LS11	Review HP	Leader reviews home practice of limit setting material	6	0.89	1.85	0.98	0.97	0.99
Effective Communication Scale								
EC1	Verbal Teach	Effective communication is a tool for exchanging information	4	0.34	.65	0.39	0.08	0.64
EC2	Verbal Teach	Characteristics of effective communication	5	0.44	.95	0.58	0.24	0.79
EC3	Verbal Teach	Characteristics of active listening	6	0.64	1.45	0.93	0.86	0.96
EC4	Verbal Teach	Active listening helps parents monitor children	5	0.52	1.15	0.84	0.66	0.93
EC5	Demonstrate	Leader demonstrates effective communication material	6	0.28	0.85	0.34	0.01	0.60
EC6	Facilitate	Leader facilitates in-session application of effective communication material	6	0.53	1.26	0.82	0.68	0.91
EC7	Assign HP	Leader assigns home practice of effective communication material	5	0.52	1.12	0.92	0.84	0.96
EC8	Review HP	Leader reviews home practice of effective communication material	6	0.70	1.52	0.87	0.76	0.93
Problem Solving Scale								
PS1	Verbal Teach	Problem solving is a tool for addressing problems and/or achieving goals as a family	6	0.43	.88	0.56	0.29	0.75
PS2	Verbal Teach	Rationale for specific problem solving procedure	6	0.55	1.37	0.89	0.80	0.94
PS3	Verbal Teach	Instructions for specific problem solving procedure	6	0.84	1.76	0.94	0.89	0.97
PS4	Verbal Teach	Rationale for family meetings	4.5	0.26	0.76	0.92	0.86	0.96
PS5	Verbal Teach	Instructions for family meetings	5	0.25	0.82	0.90	0.81	0.95
PS6	Demonstrate	Leader demonstrates problem solving material	5	0.35	1.05	0.85	0.73	0.92

PS7	Facilitate	Leader facilitates in-session application of problem solving material	6	0.69	1.68	0.98	0.97	0.99
PS8	Assign HP	Leader assigns home practice of problem solving material	5	0.45	1.08	0.93	0.87	0.97
PS9	Review HP	Leader reviews home practice of problem solving material	6	0.62	1.62	0.99	0.98	0.99
Monitoring Scale								
MO1	Verbal Teach	Monitoring is a tool for keeping children safe and/or out of trouble	4	0.32	0.79	0.88	0.78	0.94
MO2	Verbal Teach	Children need different levels of monitoring	4	0.19	0.64	0.80	0.64	0.89
MO3	Verbal Teach	Aspects of monitoring inside the home	4	0.07	0.38	-0.01	-0.34	0.31
MO4	Verbal Teach	Aspects of monitoring outside the home	6	0.46	1.22	0.84	0.71	0.92
MO5	Verbal Teach	Involving children in skill-building activities	4	0.15	0.58	0.86	0.74	0.92
MO6	Verbal Teach	Protecting children while in care of others	6	0.28	1.04	0.87	0.76	0.93
MO7	Demonstrate	Leader demonstrates monitoring material	3	0.07	0.36	-	-	-
MO8	Facilitate	Leader facilitates in-session application of monitoring material	6	0.36	1.12	0.72	0.52	0.85
MO9	Assign HP	Leader assigns home practice of monitoring material	6	0.32	1.23	0.97	0.94	0.98
MO10	Review HP	Leader reviews home practice of monitoring material	6	0.28	1.10	0.86	0.74	0.92
Positive Involvement Scale								
PI1	Verbal Teach	Clear directions support the parent-child relationship	3	0.21	0.62	0.37	0.05	0.62
PI2	Verbal Teach	Skill encouragement is a positive experience for child/strengthens parent-child relationship	6	0.55	0.94	0.41	0.10	0.65
PI3	Verbal Teach	Emotions can interfere with parent-child relationship	6	0.51	0.94	0.73	0.54	0.85
PI4	Verbal Teach	Limit setting can be accomplished without harming the parent-child relationship	6	0.66	1.27	0.40	0.09	0.64
PI5	Verbal Teach	Effective communication nourishes the parent-child relationship	4	0.33	0.76	0.84	0.70	0.91
PI6	Verbal Teach	Problem solving prevents conflict in the parent-child relationship	3	0.21	0.60	0.12	-0.21	0.43
PI7	Verbal Teach	Monitoring can benefit the parent-child relationship	3	0.09	0.38	0.66	0.43	0.81
PI8	Verbal Teach	Showing child affection	3	0.10	0.34	-0.06	-0.38	0.27
PI9	Verbal Teach	Spending time with child	2	0.22	0.45	0.26	-0.07	0.54
PI10	Verbal Teach	Being involved in child’s school life	3	0.26	0.64	0.51	0.15	0.76
PI11	Verbal Teach	Positive involvement not otherwise specified	2	0.26	0.53	-0.05	-0.37	0.28

Note: HP = home practice.
