# Supplementary material for: Development and Evaluation of a Component Level Implementation Fidelity Rating System for the GenerationPMTO Intervention
Source: Prev Sci. 2020 Oct 25;22(3):288–98. doi: 10.1007/s11121-020-01177-5 (PMC8032561; doi:10.1007/s11121-020-01177-5)
Supplement: Supplementary file 3 — (PDF 117 kb) [file 11121_2020_1177_MOESM3_ESM.pdf]

Supplemental File C

Correlations for Intervention Group Component Scores and Post-Intervention Observed Parenting Practices

CLIFRS Component Scores	Post-Intervention Observed Parenting Practices Time 1	Time 2	Time 3
		Negative Reciprocity	
Clear Directions	.00	-.21*	.04
		Skill Encouragement	
Skill Encouragement	.04	.16*	.10
		Emotion Regulation	
Emotion Regulation	.15*	.19*	.02
		Inept Discipline	
Limit Setting	-.19*	-.06	-.04
		Effective Communication	
Effective Communication	.15*	.10	-.04
		Problem Solving	
Problem Solving	.13	.12	.18*
		Monitoring	
Monitoring	.00	.18*	-.10

Note. Time 1 = 6 months post-baseline, Time 2 = 12 months post-baseline, Time 3 = 30 months post-baseline. These timepoints were selected from among five assessment occasions because they allowed for the examination of predictive validity at termination, at the first follow-up assessment, and at the most distal follow-up. Observation-based items from structured interaction tasks were used to derive variables for skill encouragement (e.g., *Mom broke down tasks as necessary*, *Mom provided reinforcement for on task behavior*); emotion regulation (e.g., *Mom expresses anger/hostility while disciplining, Mom: Openly angry/irritable/hostile - - Composed/calm/pleasant*), and effective communication (e.g., *Mom was positively responsive to child questions*, *Mom showed empathy, support*). Monitoring was calculated as mean of 1 observed item (i.e., *Mom knows what child doing on day to day basis*) and 3 items from structured interview (e.g., *Skillful in supervising child carefully during family task*, *Keeps close track of youngster's behavior outside of lab*). Variables for negative reciprocity, inept discipline, and problem solving were based on prior GenPMTO studies (e.g., DeGarmo et al., 2004).
*$p < .05$ (1-tailed).
